# When Personalization Does Not Change Care: A Critical Narrative Review and an Organizational Decoupling Framework

**DOI:** 10.3390/healthcare14193136

**Published:** 2026-09-22

**Authors:** Zelimkhan Berikkhanov, Zakhar Akulov, Miroslava Pilipenko, Maria Sukhanova, Evgeniy Tarabrin, Alexey Shestakov, Andrey Nikolaev, Aleksey Kotelnikov, Vadim Razumovsky, Milena Ivanova, Sara Nourmahal, Sergey Muraviev

**Affiliations:** Department of Hospital Surgery № 2, Sechenov First Moscow State Medical University (Sechenov University), 119435 Moscow, Russiamuravev_s_yu@staff.sechenov.ru (S.M.)

**Keywords:** patient-centred care, person-centred care, organizational decoupling, patient voice, goal concordance, shared decision making, feedback, recovery

## Abstract

Background/Objectives: Patient-centred practices do not by themselves show whether an individual’s priorities influence care. This review asks how organizational decoupling theory can inform a traceable response to those priorities and locate a loss of influence. Methods: A theory-informed critical narrative review used a purposive analytical bibliography of 63 publications. The authors’ preliminary categories, professional experience, and organizational theory guided interpretation. A source-to-claim matrix separates published findings, conceptual arguments, and proposed mechanisms. The framework was refined through comparison with literature, without independent inductive coding. Results: Three candidate mechanisms concern influence over service design, the expression and recognition of patient goals, and the organizational response to experience and outcome data. They may overlap and do not exhaust the possible explanations. A proposed loop links the goal, decision, action, outcome, and review. Its assessment distinguishes evidence of lost influence from justified plan retention and insufficient information. A hypothetical care pathway and a research programme specify how to test these distinctions while accounting for changing preferences, implementation fidelity, and documentation burden. Conclusions: The contribution is an account of where patient influence may be lost and what a considered response would involve. The three mechanisms and the order of the loop are conceptual propositions. Their reliability, explanatory value, and effects on care require independent evaluation before use as performance indicators.

## 1. Introduction

Patient-centred and person-centred care frameworks call for attention to people’s values, circumstances, and participation [1,2,3,4,5]. Organizations shape how those priorities are expressed through the questions they ask and the options they record. If a form offers no way to explain a concern, a completed response may leave its meaning unclear. The organizational question is whether information about a particular person affects the options offered, the decision taken, and the care delivered. Respectful communication and completed questionnaires can contribute to this process, but their presence alone does not establish that influence.

The related concepts have substantial common ground, but they are not interchangeable. Patient-centred care addresses the person receiving care, including the clinical relationship and coordination. Person-centred care places greater emphasis on the person’s life, identity, and circumstances beyond the patient role. Both can include shared decisions and organizational change [1,2,3,4,5,6]. We use personalization as a working term for the adaptation of care to an individual within these broader approaches. This use does not replace the definitions adopted by the cited studies. The ethical premise is that patients should have truthful information and an opportunity to influence decisions that concern them [7].

A healthcare organization may adopt a personalization policy, introduce a questionnaire, and train staff while retaining its previous allocation of time, authority, and resources. These activities could increase without strengthening the connection between a person’s priorities and care. Organizational decoupling refers to a separation between formally adopted policies and their implementation, or between implemented practices and the outcomes they are intended to produce [8,9]. In this sense, formal adoption or implementation does not by itself establish that an organizational practice has achieved its intended purpose. Healthcare studies identify difficulties in implementing shared decision making, barriers to collective participation, and limited evaluation of changes prompted by patient feedback [10,11,12,13]. Together, these observations provide a basis for examining where the connection between formal commitments to personalization and their translation into care may weaken.

A concern can also remain unexpressed. A person who depends on staff or access to treatment may not feel able to disagree with a proposed plan. Studies report fear of appearing difficult, reluctance to disturb staff, and uncertainty about the response to speaking up [14,15,16]. An invitation to speak therefore needs to be considered alongside the relationship in which it is offered. Silence alone cannot establish consent or the absence of an unmet concern.

A further concern is the response to measurement. Patient-reported experience measures (PREMs) describe care experiences, whereas patient-reported outcome measures (PROMs) assess health from the patient’s perspective. Feedback can improve communication and some health outcomes, although effects vary by measure, intervention, and setting [17,18,19,20,21,22]. The question for this review is how a result becomes a considered response in the care pathway. An explicit allocation of responsibility and a plan for reassessment are proposed features of that response, rather than proven prerequisites for every beneficial use of feedback.

Critiques of person-centred care identify bureaucratic constraints, conceptual tensions, and the risk that the language of centredness outpaces practice [23,24,25]. These critiques leave a practical question: Where can patient influence be lost, and what observations would distinguish that loss from responsive care? A useful organizational account must be able to recognize successful consideration as well as failure.

In this review, personalization means the capacity to adapt decisions, support, and care pathways to a person’s goals, condition, and capabilities over time. This is the authors’ analytical definition, not a consensus definition or an outcome established by the review. Adaptation can include maintaining an existing plan when it remains appropriate after discussion. It can concern care arrangements and the conditions of recovery as well as treatment. Molecular stratification is outside the present organizational focus, although some definitions of personalized healthcare include both biological and relational dimensions [26].

The primary research question is how organizational decoupling theory can help explain a loss of individual patient influence and inform a traceable organizational response. We ask where influence might be lost, what would distinguish that loss from justified plan retention, and how the proposed explanations could be tested. The practical aim is to connect what matters to a person with decisions, delivery, and review, while keeping clinical responsibility and coordination with the team. The selected literature is not intended to establish a universal definition of personalization or estimate how often it fails, but to support the development and critical examination of this framework.

## 2. Materials and Methods

### 2.1. Design and Scope of the Review

We conducted a theory-informed critical narrative review to develop and critically examine an organizational argument. Preliminary categories, professional experience, organizational decoupling, and the working definition in the Introduction supplied the interpretive frame. Literature supported, qualified, or challenged its components. This design fits an explanatory and partly normative question across heterogeneous literatures [27,28,29]. Concept analysis would make the definition of personalization its primary object. A scoping review would systematically map the field. Critical interpretive synthesis could support more formal iterative theory development, but its procedures were not implemented here. The present work is therefore explicitly theory-informed, without claiming independent derivation of a taxonomy.

No prospective protocol, independent duplicate screening, or formal study-level risk-of-bias assessment was used. A two-stage review could first map definitions and then examine decoupling. Here, conceptual clarification supports the narrower organizational question within one narrative argument. These are logical steps, not two prospectively conducted review phases. Because the initial framework influenced selection and interpretation, circularity and confirmation bias remain possible. Within these limits, the chosen design preserves the intended focus on decisions, support, and the conditions of recovery.

### 2.2. Information Sources and Search Logic

The literature was assembled during July and early August 2026 through targeted PubMed/MEDLINE searches, Crossref metadata checks, reference list reading, forward citation following, and journal websites. Crossref served bibliographic retrieval and verification rather than comprehensive subject searching. Database-specific search histories and intermediate retrieval counts were not retained.

Thematic searches addressed centredness, organizational implementation, voice and dependency, measurement and feedback, shared decisions, coproduction, recovery, and value. Table S1 provides complete illustrative PubMed expressions for these themes and distinguishes them from the limited query examples retained in the preparation text. The expressions are reporting aids prepared for this revision. They were not a prospectively specified strategy and are not presented as the exact queries originally executed. Figure S1 shows the reported retrieval routes and the traceable analytical reference set without invented screening counts.

Foundational conceptual and methodological works were retained alongside more recent syntheses and empirical studies. No fixed publication-year rule or date filter can be established from the retained material. The cited literature includes reviews published in 2024 and 2025. Its currency should be read in relation to this purposive selection, not as evidence of an exhaustive search through the revision date.

### 2.3. Corpus Assembly and Selection Criteria

The analytical bibliography in this revision comprises 63 publications, all mapped in Table S2. It includes the 62 references in the submitted manuscript and one Cochrane review already present in the background reading collection [22]. The earlier figure of 105 full texts referred to that broader reading collection, rather than 105 separately documented contributions to the final argument. Neither that figure nor the preliminary tally of 111 records is retained as a screened-corpus denominator. Without source-level selection records, their difference cannot be presented as a reproducible exclusion count. The source matrix provides an auditable account of the works actually used to support this review.

Sources were selected for their relevance to the conceptual question. They address definitions of centredness, organizational conditions, the expression of priorities, participation in decisions, the use of patient-reported information, meaningful outcomes, or resource consequences. Conceptual papers, reviews, qualitative studies, observational research, and intervention studies could contribute. Methodological publications support the review design. A source did not have to document the complete proposed loop or a failure of personalization to be informative. Accounts of successful implementation and positive feedback effects constrain the argument. Genomic stratification without an organizational connection and preclinical or animal research do not support the present framework. These statements describe the rationale of the selected bibliography, not a recovered protocol of record-by-record eligibility decisions.

### 2.4. Conceptual Development and Source Mapping

The author group selected and interpreted publications. S.M., Z.A., and M.P. contributed to the methodology. Z.A., M.P., M.S., E.T., and Z.B. contributed to the formal analysis, and Z.A., M.P., Z.B., V.R., M.I., and S.N. to the data curation. Source-level allocations of selection and extraction were not recorded. Independent duplicate coding was not conducted. Interpretive differences were discussed with reference to the source, without a retained disagreement log or formal adjudication. Table S2 maps each cited work’s design, argumentative role, and limits for this revision. It is a retrospective source map, not a recovered standardized extraction record.

The three candidate mechanisms and the loop were formulated provisionally before the present synthesis, drawing on professional experience and preliminary reading. Comparison with the literature refined their wording and scope. Four analytical steps organize this account. First, the conceptual literature clarifies related terms while preserving their differences. Second, organizational decoupling supplies a lens for examining separation from practice or intended outcomes. Third, findings on service design, voice, and feedback are considered alongside successful implementation and alternative explanations. Fourth, the preliminary categories and loop are qualified and translated into testable propositions. These steps describe the argument, not independent inductive coding. Table S3 links findings and counterarguments to the resulting qualifications.

The formulation is also informed by the authors’ clinical and organizational experience. Experience helped frame questions about what organizations regard as a completed response. It was not collected or coded as study data and cannot establish feasibility or effectiveness. The framework therefore remains open to counterexamples, including successful care delivered through arrangements that differ from the proposed loop.

### 2.5. Status of the Evidence and Control of Extrapolation

We distinguish four types of statement. Empirical findings retain the design and population of the source. Conceptual arguments include the authors’ working definition and explanations informed by theory and professional experience. The three mechanisms, the loop, and its operational signals are hypotheses. Normative commitments concern truthful explanation, clinical responsibility, and equitable access. Table 1 separates evidence close to a component from broader conceptual support. Neither validates the integrated framework. Table S2 specifies each publication’s role and limits, including findings that restrict the argument. Evidence from intensive care, serious illness, geriatrics, palliative care, and mental health remains bounded by those settings.

No common quality scale was applied to this heterogeneous bibliography. Source relevance, methodological transparency, and limits of transfer were considered narratively, without a formal weighting score. Reviews and reports from the same programmes can overlap, so their findings are not counted as independent replications. In particular, the Veterans Health Administration studies [30,31] draw on related transformation work, and the PROM reviews share primary studies [18,19,20,21,22]. Quantitative results are retained only with their study context. There is no pooled estimate of personalization or organizational decoupling.

### 2.6. Quality Control Using SANRA

SANRA was used as an editorial checklist covering importance, aims, search description, referencing, scientific reasoning, and presentation of relevant data [47]. It was not used to assess individual studies or generate the conceptual categories. No total score or claim of independent quality certification is made. The checklist does not remedy missing search records or selection bias.

## 3. Conceptual Synthesis

### 3.1. From a Broad Principle to a Testable Organizational Property

Patient-centred care includes recognition of the patient as a person, a biopsychosocial perspective, shared influence, and the therapeutic relationship [1,2,4]. Person-centred care gives additional emphasis to the person’s life and identity beyond a care episode, while also addressing structures, processes, and outcomes [3,5,6]. These are overlapping orientations with variable definitions across disciplines. Neither is reduced here to politeness or satisfaction.

Shared decision making concerns how patients and clinicians consider evidence, options, and preferences together [48,49]. Coproduction concerns their contribution to producing care through their interactions [50,51]. Collective co-design addresses the development of services for groups, which is a different unit of participation from an individual treatment decision [11,52]. Patient voice refers here to the expression of a person’s experience, priorities, or dissent. Patient-reported information also includes structured measures that need not preserve that person’s own formulation [12,13,14,15,16,17,18,19,20,21,33].

Our working definition asks whether an individual’s priorities influence decisions, care delivery, and review. A priority is what the person identifies as important at that time. It can concern a desired outcome, a treatment preference, or the conditions of care. A goal gives an intended direction to planning, while a decision records what the team and patient will do or retain. Delivered action and its outcome must then be assessed separately. These working distinctions do not require a different treatment for every patient. A standard plan can remain appropriate after the person’s position has been considered and its rationale explained.

### 3.2. The Architecture of the Evidence and the Limits of Inference

The supporting literature differs in design, setting, and proximity to the question. Organizational studies address implementation, while research on voice and feedback examines particular relationships and processes. Recovery and capability literature broadens the interpretation of outcomes. Economic studies evaluate specific interventions or associations. These contributions are related analytically, without assuming that all sources study the same construct (Table 1). Table S2 identifies the contribution of every cited publication.

The three candidate mechanisms and the loop are the authors’ conceptual construction. Published findings support or challenge components of that construction. No included study validates the framework as a whole, establishes the necessity of all five links, or confirms their proposed order.

### 3.3. The First Decoupling: Representation Without Influence on Design

The first candidate mechanism concerns how organizations define the available field of care. Appointment time, referral options, professional authority, and resource rules can restrict what an individual clinician is able to offer. Organizational theory distinguishes formal policy that remains detached from practice from implemented procedures whose connection to their intended ends is weak [8,9]. Applying that distinction to personalization directs attention to whether patient contributions influence the design and resourcing of care.

Qualitative studies in US organizations selected for their commitment to patient-centred care describe leadership, patient and staff engagement, changes in roles, and sustained resourcing as facilitators of implementation [30,32]. Related Veterans Health Administration research found both broader organizational understandings and more traditional interpretations of patient-centred care among staff [31]. These studies support attention to working conditions and organizational culture. They do not isolate leadership as a sufficient cause of success or establish the effect of the present framework.

Representation without influence is proposed when patient contributions enter a formal participation process but are not substantively considered in the design of services. Collective participation may influence quality criteria, care options, or resource allocation, yet an invitation to participate does not establish that such influence occurred. A systematic review identified persistent barriers to meaningful collective participation [11]. A critical review likewise drew attention to unequal power and tokenism [52]. A reasoned decision not to adopt a proposal is different from excluding it from consideration.

Aggregate ratings and representative bodies cannot stand in for each person’s priorities. Collective input may change which options exist, but those options still need to be considered with the individual. This distinction matters in multimorbidity, where clinicians report competing goals, limited time, and difficulties coordinating care [53]. The proposed connection between collective design and individual care must therefore be traced through implementation and use. It cannot be inferred from the existence of a patient council.

Standardization can support safety and continuity. The candidate decoupling concerns an inability to consider a relevant individual priority within available options, or to seek an authorized review when the standard pathway is unsuitable. Evidence of the mechanism requires more than an unchanged plan. A researcher would need to examine the patient’s contribution, the decision process, the available authority, and the explanation for retention or change.

### 3.4. The Second Decoupling: Need Without Effective Voice

Relying on patients to initiate every discussion makes their concerns dependent on their ability to speak. A person may need help to describe what matters or may doubt that their account will be taken seriously. Carel and Kidd discuss this problem through epistemic injustice, including the discounting of patient testimony and difficulties making illness experience intelligible to others [54]. This philosophical argument provides an interpretive lens. It does not establish how often such a loss occurs or whether it explains a particular silence.

Studies of voice identify several reasons why a patient may hesitate to object. In focus groups, participants described deference to physicians and fear of being labelled difficult [14]. A qualitative study across care settings found that speaking up about safety depended on the concern, relationships with staff, and the anticipated response [15]. In intensive care, patients and family members reported reluctance to disturb staff or appear troublesome [16]. These findings suggest possible constraints on expression. Their frequency and relative importance cannot be transferred to routine outpatient, surgical, or other national settings.

A vignette-based randomized survey found that an explicit invitation to report breakdowns increased stated willingness to speak up [55]. It tested a response to a scenario, not actual disclosure or improved care. The organizational implication proposed here is to make it possible to express and revisit concerns, including in a private conversation when desired. The safety of that opportunity needs to be assessed from the patient’s perspective rather than assumed from the wording of a question.

Agency includes the ability to understand options, form a preference, and act with appropriate support. Entwistle and colleagues argue that relationships can enable autonomy rather than simply constrain it [56]. A systematic review of patient-reported barriers to shared decision making similarly identifies knowledge and power as relevant to participation [33]. These accounts support attention to the conditions for influence. They do not require patients to choose unaided or rule out freely chosen delegation.

The second candidate mechanism concerns a loss between a person’s position and its recognition in decision making. Silence, a signed consent form, or the absence of complaints cannot establish whether that loss occurred. A difference between routine and protected interviews is a signal for investigation. It may reflect earlier restraint, but also new information, a changing preference, or an interviewer effect. Evaluation must establish when the preference formed and why the account changed. Conversely, an unchanged response may indicate that the initial consultation was already safe.

### 3.5. The Third Decoupling: Measurement Without Action

Satisfaction, experience, and outcome measures answer different questions. Satisfaction depends partly on expectations and context, so it cannot by itself establish technical quality or concordance with a person’s goals [57]. A systematic review found positive associations between patient experience, safety, and effectiveness [17]. The concern here is therefore how a measure is interpreted and used. An indicator should not be assumed to capture every aspect of the care it describes.

In a US observational cohort, higher satisfaction was associated with greater inpatient use and expenditure [58]. This does not show that satisfaction causes poor outcomes. Confounding, reverse causation, and differences in expectations remain possible. The finding limits the use of satisfaction as a standalone indicator of value, without negating its relevance to patient experience.

Reviews of patient experience data show that collecting information and improving care are different tasks. Gleeson and colleagues found that reported improvements often concerned appointment arrangements and information materials, with limited evaluation of their effects [12]. Gilmore and colleagues identified PREM use at several system levels but limited evidence that organizational- or system-level measurement produced evaluated improvements at the point of care [13]. Bull and colleagues found variable evidence for the measurement properties of PREMs [34]. These findings question unsupported assumptions about implementation, rather than the value of measuring experience itself.

PROM feedback has a more mixed and partly positive evidence base than a simple account of measurement failure would suggest. An early systematic review found inconsistent benefits [18]. A 2024 overview of 40 reviews found clearer effects on care processes than on health outcomes and generally low review quality [19]. A Cochrane review of 116 randomized trials found moderate-certainty evidence of small quality-of-life improvement, with a standardized mean difference of 0.15 (95% confidence interval 0.05 to 0.26) across 11 trials involving 2687 participants [22]. All included trials were from high-income countries. Other reviews suggest greater benefit when PROMs support monitoring and a clinical response, although these comparisons do not isolate a mandatory response architecture as the cause [20,21]. These overlapping reviews cannot be treated as independent replications.

The third mechanism must therefore be framed conditionally. It concerns relevant experience or outcome information that receives no substantive consideration when a response is indicated. A named recipient, a locally appropriate response rule, and reassessment are proposed ways to make that consideration observable. A clinically justified decision to continue the plan can complete the response. No universal numerical threshold, staffing arrangement, or review interval follows from this literature.

Goal-concordance research offers ways to examine the correspondence between priorities and care. A 2025 scoping review in geriatrics and palliative care identified 44 unique measures within 63 studies [35]. Its conceptual model distinguished the preferred outcome or treatment, treatment received or intended, alignment with preference, and achievement of the desired outcome. These are related but distinct properties. Methodological work cautions that recall, timing, and population-level proxies can distort their assessment [36]. In a secondary analysis of baseline surveys from a US trial, 58% of 405 seriously ill outpatients reported goal-concordant care, 17% reported discordance, and 25% were uncertain of its focus [37]. Those figures illustrate measurement in that population. They do not estimate the prevalence of the proposed decouplings.

The third candidate decoupling arises when patient-reported information is recorded but does not enter a considered decision, action where indicated, and review. Questionnaire completion cannot establish whether these steps occurred. The analysis requires a link to the actual response and its rationale. Missing documentation leaves that link uncertain until other evidence is considered.

### 3.6. A Framework of Organizational Decoupling in Personalization

We group the proposed losses of influence by the organizational task they concern. Design addresses available options and patient contributions to their development. Expression and recognition address whether an individual’s position can be expressed and substantively recognized in decision making. Response addresses what happens after relevant information reaches the team. This functional grouping suggests different targets for investigation, including authority to adapt services, conditions for speaking, and responsibility for follow-up. It does not establish three independent causes, their relative prevalence, or their completeness.

The categories may overlap without being equivalent. Adaptable service options can coexist with a patient’s fear of expressing a preference. A team can recognize that preference but fail to respond to a later outcome signal. A classification by structure, process, relationships, and culture is a plausible alternative, as is one by patient, clinician, and organization or by micro-, organizational, and system levels. Those approaches answer different questions and have not been shown to be inferior. Financial incentives, interprofessional conflict, patient–family disagreement, and conflicts between local practice and national requirements may cut across the proposed categories or warrant additional ones. Comparative testing should permit multiple labels and an unclassified category. Each assigned mechanism requires its own evidence. The proposed mechanisms and the evidence needed to interpret them are summarized in Table 2.

For all three mechanisms, an unchanged plan is not evidence of failure. Code insufficient evidence when records and corroborating accounts do not establish whether a priority was considered. Code a supported loss only when relevant evidence indicates that influence was lost. Justified retention and clinically constrained choices require a traceable rationale, but do not require the patient to agree with every limitation.

The proposed loop connects five analytical links: goal, decision, action, outcome, and review. Goal elicitation includes joint clarification and the person’s desired degree of participation. The patient’s wording and the agreed interpretation should remain distinguishable. Decisions may retain or alter the plan. An assigned task is not delivered action, and a completed record is not an achieved outcome. Reviews consider clinical, functional, and personally meaningful results, which need not move together. This sequence is a design logic. In practice, links may recur, overlap, or follow another order, and urgent care may precede discussion. Shared decision-making models already include deliberation and follow-up [48,49]. The proposed addition is to trace influence through organizational delivery and review.

Coproduction also extends beyond a single choice by recognizing that care is produced through patient–professional interactions [50,51]. The present framework draws attention to possible losses across those interactions. It does not claim to replace coproduction, shared decision making, or existing person-centred frameworks. Collective service design can make an option available. Individual deliberation determines whether that option fits this person. Organizational processes determine whether it is delivered and reviewed. Evidence at one of these levels cannot substitute for evidence at another. These distinctions and the proposed loop are summarized in Figure 1.

Panel A distinguishes published findings from the authors’ conceptual propositions. Panel B presents five links and three candidate mechanisms. Their order is an analytical proposal, and mechanisms can co-occur. Panel C distinguishes supported loss of influence, justified retention, and insufficient evidence. Plan change is not a success criterion by itself. The figure is a research framework whose categories and sequence require independent testing.

#### A Hypothetical Pathway and Counterexample

Consider a hypothetical patient preparing for discharge after elective surgery who wants to recover at home but is uncertain about managing the care required there. A patient council has recommended clearer discharge support, but no service option or responsible role has been established. This could illustrate a design problem if the contribution was not substantively considered. In a private conversation, the patient says that the uncertainty existed before the ward round but was not raised for fear of delaying discharge. That account raises a possible voice mechanism. If the concern is then recorded and reaches the team but receives no consideration or follow-up, a response mechanism may also be present. These are stipulated features of an illustration, not observations from an actual patient or evidence that all three mechanisms usually co-occur.

A contrasting pathway could retain the same discharge date. The team explores the patient’s goal, assesses what support is needed, explains the available arrangements, and agrees on a feasible plan with a named contact and review. The date remains unchanged because the concern has been addressed, not because it was ignored. If all that is available to an assessor is the phrase ‘patient informed’, neither version can be established. That episode should be coded as insufficient evidence until the account of the discussion and care delivered can be checked. Failure to improve a clinical outcome would likewise not, by itself, prove that the patient’s priorities were disregarded.

### 3.7. Treatment, Outcome, and Recovery

Closing the proposed loop requires distinguishing treatment, its clinical result, and its meaning for the person. An intervention may be technically appropriate while leaving an important functional problem unresolved. A clinical improvement may not restore the ability to work or care for others. In incurable disease, relief, adaptation, and a meaningful life may remain achievable without return to a previous state. Biopsychosocial and quality of life models relate biological variables, symptoms, function, perceived health, and context [38,40,41]. They support assessing these dimensions separately rather than treating any one as a complete outcome.

Recovery, in this work, is not set in opposition to treatment. It denotes a broader horizon within which the clinical result is related to the possibility of living a life that is meaningful to the person concerned. Conceptualizing health as the ability to adapt and manage one’s condition makes it possible to discuss this horizon in chronic illness and disability [59]. The capability approach shifts attention from the formal right to choose to a person’s real capacity to act [60,61].

The literature on suffering and on personal recovery explains why an identical clinical outcome can carry different meanings. Disruption of the integrity of the person, of their biography, and of their relationships is not exhausted by the severity of a symptom [62,63]. In mental health, recovery processes encompass connectedness with others, hope, identity, meaning, and greater influence over one’s own life. Transferring this model to other fields requires verification. What matters for our frame here is the possibility of recovery while illness continues [39].

### 3.8. Value and Economic Consequences

Porter’s value framework relates health outcomes to the costs of the full cycle of care [42]. This conceptual relation does not predict automatic savings from personalization. A systematic review found more consistent associations of patient-centred care with satisfaction and self-management than with clinical outcomes [43]. An observational primary-care study associated a patient-centred style with lower subsequent service use and charges, but could not establish causation [44].

Specific interventions provide more direct, context-bound evidence. A one-year US randomized study of enhanced remote decision support reported lower medical costs, admissions, and preference-sensitive operations than usual support [45]. A systematic review and meta-analysis of self-management support found potential reductions in service use without deterioration in outcomes, with substantial variation by intervention and condition [46]. Neither intervention is equivalent to the organizational framework proposed here.

Economic effects remain hypotheses for this framework. Possible pathways include less discordant or low-value care, earlier recognition of problems, improved coordination, and sustained self-management. Each requires separate measurement. Changes in access, outcomes, and unpaid family work must be assessed alongside organizational costs. Savings alone cannot establish that care became more responsive to a person’s priorities.

### 3.9. Testable Propositions

Table 3 presents research hypotheses. Their assessment requires reliable measurement, a meaningful comparison, and uncertainty estimates. A statistically non-significant result is not evidence of equivalence. Conversely, a precise estimate that excludes a prespecified meaningful benefit can weaken a proposition within the setting studied. Replication would strengthen that challenge, but is not required before an informative negative finding is acknowledged.

## 4. Discussion

### 4.1. The Principal Finding and Its Significance

This review proposes a way to locate a loss of patient influence within care. Its contribution is the distinction between the design of available options, the conditions of expression, and the response to information. Although the working definition limits what the framework can explain, consistency between that definition and the selected literature should not be interpreted as independent validation. Within these boundaries, the definition makes the proposed points of lost, preserved, or uncertain patient influence explicit and open to empirical investigation.

The synthesis answers the research question by identifying candidate points for observation rather than establishing causes or prevalence. Existing models already address structures, processes, and outcomes [3,4], deliberation [48,49], and coproduction [50,51], and the present framework does not claim superiority over these approaches. Its proposed contribution is to connect organizational decoupling to individual care through decisions and actions that can be examined over time, providing explicit distinctions and test conditions for investigating when patient influence is lost, preserved, or impossible to determine.

### 4.2. Relationship to Existing Evidence and Alternative Explanations

Organizational decoupling theory distinguishes a policy that is not implemented from an implemented procedure whose relationship to its intended outcome is uncertain [8,9]. Our three categories do not replace that distinction. They locate potential losses within personalization-related work and can include either kind of decoupling. A gap does not establish deliberate simulation. Competing requirements, scarce resources, uncertain causal links and weak coordination may produce similar observations. The framework should help investigate these explanations rather than assign motives.

Successful implementation provides an important counterargument to an account of inevitable failure. Studies describe sustained leadership, patient and staff involvement, role alignment, and resources for redesign [10,30,31,32]. Positive effects of PROM feedback also show that measurement can benefit patients [20,21,22]. These findings restrict the argument to circumstances in which relevant influence is lost. They do not establish that all successful organizations use the proposed loop or that each proposed component is necessary.

Effective voice also depends on the degree of participation a person wants. Fear, deference, or dependence may inhibit expression [14,15,16,33]. Silence and delegation nevertheless cannot be classified as lost agency without further evidence. A person may choose to let the clinician lead, need temporary support, or prefer to revisit a decision later. Relational autonomy and shared decision making support giving attention to that preference and the opportunity to reconsider it [48,49,56]. Protected elicitation remains a proposed research technique. A change in the account requires explanation before it is attributed to earlier suppression.

Measurement without a documented change is especially easy to misclassify. The plan may already match the patient’s priorities, or a requested option may be clinically unsuitable. A preference may have changed. Alternatively, discussion and action may have occurred without adequate recording. Table 2 therefore requires corroborating information and a decision rationale. Substantive consideration can result in retained treatment, an adapted support arrangement, or a justified limitation. A completed field, an unchanged pathway, and a favorable outcome are each insufficient to decide which process occurred.

Goal-concordance research shows that such comparison is possible while exposing its measurement limits [35,36,37]. There is no single reference standard. Recall, treatment outcomes, and the interval between a stated goal and care can affect the account. Population measures can also conceal minority preferences. Testing the framework therefore requires linked information about the priority, decision, delivered action, outcome, and grounds for review. These data need dates and context, rather than an assumption that a complete record captures influence.

### 4.3. Managerial Implications and the Russian Context

The framework directs managerial attention to authority, time, and responsibility. Leaders influence which patient concerns receive consideration, who can authorize a response, and what counts as completed work. These decisions give organizational commitment an observable form. Implementation studies support attention to leadership, roles, and resources [10,30,31,32]. They do not establish that commitment alone can overcome constraints or that the proposed arrangements will work in every setting.

A practical implication is to seek priorities early enough to inform planning, then assess whether the response addressed them. This places the patient’s contribution before, as well as after, organizational action. It preserves clinical assessment and responsibility, including necessary urgent treatment. Recovery, function, and relief may be personally meaningful aims [39,40,62]. Patient-reported priorities and clinically identified needs should be discussed together. Clinical signs can prompt assessment, but neither silence nor a physiological sign establishes a person’s unspoken preference.

An initial evaluation could use a brief priority statement, a named responsible professional, the rationale for the response, and a review linked to a relevant clinical transition. These are candidate arrangements, not staffing or documentation standards. The team remains responsible for coordination, including when a patient delegates decisions or cannot participate. Measurement should separate actual care from record completion and include time spent documenting, responding, and obtaining clarification. Resource limits may constrain even a carefully designed approach. Universal claims of low cost or feasibility are not supported [42,43,44,45,46].

The literature does not establish the prevalence or distribution of these mechanisms in Russia. Comparative research could examine how financing, referral authority, and organizational rules affect which patient contributions become usable care options. Preferred family involvement, language support, and perceived dependence may affect whose account is heard. Transfers between teams may affect who receives a signal and can authorize a response. These are proposed explanations to investigate across systems and cultures, not established national profiles. Individual preferences about family participation should be elicited rather than assigned from cultural membership.

The relevant transitions also differ by specialty. Chronic care permits repeated contact but may lose continuity between visits. Elective surgery connects preparation, admission, and recovery after discharge. In intensive care, incapacity and urgency may require supported or proxy participation and later clarification. Palliative care raises questions about changing goals and the balance of symptom relief, function, and burden. These examples identify where to observe the proposed links. They do not establish a ranking of vulnerability or prescribe one timing for all pathways.

Organizational size may modify the proposed mechanisms in opposing ways. Familiarity in a smaller institution could help staff notice a concern, but may also make private dissent difficult. A large centre may offer more alternatives while making responsibility harder to follow across teams. These hypotheses draw on the organizational argument and professional experience. They call for comparison of actual processes and resources. Size or technology alone cannot establish whether care is personalized.

### 4.4. Strengths and Limitations

This review brings organizational theory into dialogue with research on interaction, feedback, recovery, and value. Its source matrix exposes where empirical findings support an argument and where interpretation begins. Positive studies and alternative explanations restrict the proposed mechanisms, and the hypotheses specify observations that could challenge them. SANRA supported editorial review [47]. These features improve the transparency of the argument without converting purposive selection into a systematic evidence base.

Selection and interpretation were guided by the authors’ working definition and prior theory. Independent coding, a prospective protocol, a complete search history, and a source-level exclusion log are unavailable. The matrix assembled for this revision cannot recover those procedures or remove confirmation bias. The broader reading collection must not be treated as a denominator for estimating effects or completeness. The analytical bibliography can omit relevant or contrary literature, and its overlapping reviews do not supply independent replication. No prevalence estimate, causal ranking, or pooled clinical effect is warranted.

Transfer is another limitation. Much of the empirical evidence comes from high-income systems. Intensive-care studies illuminate dependency under acute vulnerability, while goal-concordance instruments and personal-recovery frameworks were developed for particular populations. Linguistic, cultural, and organizational equivalence cannot be assumed [16,22,35,36,37,38,39]. The authors’ professional experience, including S.M.’s clinical and managerial work across organizations of different sizes, informed interpretation but was not analyzed as an empirical dataset. The hypothetical example supplies no original patient evidence. Neither experience nor an illustrative case validates the framework.

The framework may also mistake traceability for substance. A complete record can conceal an unconsidered priority, while a sparse record can accompany responsive care. Retrospective accounts may rationalize earlier decisions, and repeated questions can alter the priorities they are intended to measure [35,36]. The five links may not be necessary in every episode, and review can lead to justified retention. These limits should be tested alongside alternatives to the three-category structure. Additional documentation can itself become a burden and separate measured compliance from care.

### 4.5. Priorities for Empirical Testing

Empirical work should first establish whether the proposed distinctions can be assessed reliably. A dated manual should define each mechanism, justified retention, and insufficient evidence. Two assessors could independently code records, consultation observations or recordings, and patient interviews. Report agreement for each mechanism before adjudication, with uncertainty intervals, co-occurrence, and unclassified cases. Keep training cases separate from the reliability sample. Comparisons across languages, specialties, and organizations should first examine whether the same criteria capture comparable processes. Table S4 provides candidate rules. The proposed measurement and testing programme is summarized in Figure 2.

The programme links evidence about the patient’s priorities to decisions, delivered care, outcomes, and review. Record completeness is assessed separately from patient influence. Reliability, alternative explanations, implementation fidelity, and workload require assessment before causal or comparative conclusions are drawn. The stages are proposed research tasks, not completed studies or mandatory practice standards.

An optional development task is to build a cumulative question bank from patients’ own formulations. Patients should help select and test the wording, while open responses preserve concerns absent from the existing bank. This would organize elicitation rather than produce a satisfaction score. Content validity, usability, and the stability of any proposed clusters would require evaluation on new material. Grouping concerns could support service planning, but a group profile cannot determine an individual’s priority. The bank would not itself measure decoupling or replace validated PREMs and PROMs. Existing records and focused sampling should be considered before adding routine forms.

A prospective programme should address four tasks:

- Develop and test mechanism-specific coding rules, preserving the distinction between insufficient evidence and supported absence of a mechanism.
- Link each selected patient priority to the decision, action, outcome, and review, while recording when the priority arose or changed.
- Compare interventions with suitable controls, including repeat interviews without an added safety intervention and feedback delivered with or without a specified organizational response.
- Prespecify meaningful benefits and harms, including goal concordance, clinical and functional outcomes, access, safety, equity, staff time, and work borne by patients or families.

Negative findings must remain capable of changing the framework. Stable answers after a protected interview are inconclusive if the initial encounter was already safe. Changed answers require examination of timing, new information, and interviewer effects. Feedback comparisons should verify delivered responses in both groups and account for observation-related attention, case mix, and missing data. Confidence intervals for the between-group contrast should be interpreted against a prespecified meaningful benefit. Table 3 sets out these distinctions. A precise null or contrary result can weaken a proposition in the setting studied. Replication tests whether that challenge transfers to other settings.

### 4.6. Conceptual Risk: The Managerial Model and Patient Well-Being

Alongside the methodological limitations discussed above, the proposed conceptual framework carries a different kind of risk, substantive rather than procedural. A shift in emphasis towards organizational mechanisms—authority, roles, allocation of responsibility, documentation, and feedback—may inadvertently move attention away from a person’s recovery and well-being towards care delivery as a technical process. Under such an interpretation, personalization risks becoming a set of procedures assessed by formal indicators of completion rather than by whether the patient is actually better off. Care may remain technically appropriate while losing its connection to what the person considers important in their life.

This risk is not an inevitable consequence of organizational analysis. Its possibility, however, means that the proposed framework should be assessed not only by how well it describes candidate points at which influence may be lost, but also by whether it ultimately contributes to patient well-being rather than merely to completion of the organizational loop. The distinction between service delivery as an end in itself and as a means remains open to empirical testing. It should be considered when interpreting any observed improvements, including those that formally demonstrate the traceability of a priority but do not establish its significance for the person.

The proposed loop—goal, decision, action, outcome, and review—retains value only insofar as outcome and review are related to what the person regards as well-being, rather than solely to the fulfilment of organizational requirements. Recovery, function, symptom relief, and the ability to live a meaningful life cannot be replaced by indicators of process completion. The priorities for empirical testing outlined below should therefore include not only assessment of the reliability of the proposed distinctions and the reproducibility of the mechanisms, but also examination of whether traceable organizational influence leads to changes that are meaningful to the patient.

## 5. Conclusions

This review proposes a way to conceptualize where the influence of an individual patient may be lost within care and specifies conditions for distinguishing such loss from justified retention of an existing plan and from insufficient evidence. The principal contribution is the distinction between three candidate mechanisms—the design of available options, the conditions for expressing the patient’s position, and the response to information received—and the description of five analytical links in the loop: goal, decision, action, outcome, and review. These propositions constitute the authors’ conceptual framework rather than established causal relationships or an empirically validated taxonomy.

The proposed framework does not replace existing concepts of patient-centred and person-centred care, shared decision making, or coproduction. Its contribution is to make candidate points of lost influence observable and to specify conditions under which they can be tested. None of the proposed mechanisms should be regarded as established; their prevalence, relative importance, and explanatory value remain open to independent evaluation.

A further substantive limitation arises from the organizational orientation of the framework itself. Shifting attention towards managerial mechanisms may result in patient well-being being replaced by formal indicators of service completion. In that case, personalization risks becoming a technical element of an organizational process, losing its connection to what the person considers important for their life and recovery. This possibility does not invalidate the proposed framework, but it imposes an additional requirement: any observed improvements should be assessed not only in terms of traceability, but also in terms of their significance to the patient. The distinction between service delivery as an end in itself and as a means remains fundamental.

This review cannot establish the prevalence of the proposed mechanisms, their causal hierarchy, or a pooled clinical effect. Literature selection was purposive and guided by the authors’ working concept; independent coding, a prospective protocol, and a complete search history are unavailable. Much of the empirical evidence comes from high-income healthcare systems and specific clinical contexts, which limits transferability. The authors’ professional experience and the hypothetical example do not validate the framework.

Future work should focus on independent testing of the proposed distinctions, the development of reliable coding approaches, and assessment of whether traceable organizational influence leads to changes that are meaningful for patient well-being. Until such evidence is available, the three mechanisms and five links of the loop should be regarded as conceptual propositions requiring empirical validation rather than as performance indicators or standards of practice.

## Figures and Tables

**Figure 1:**
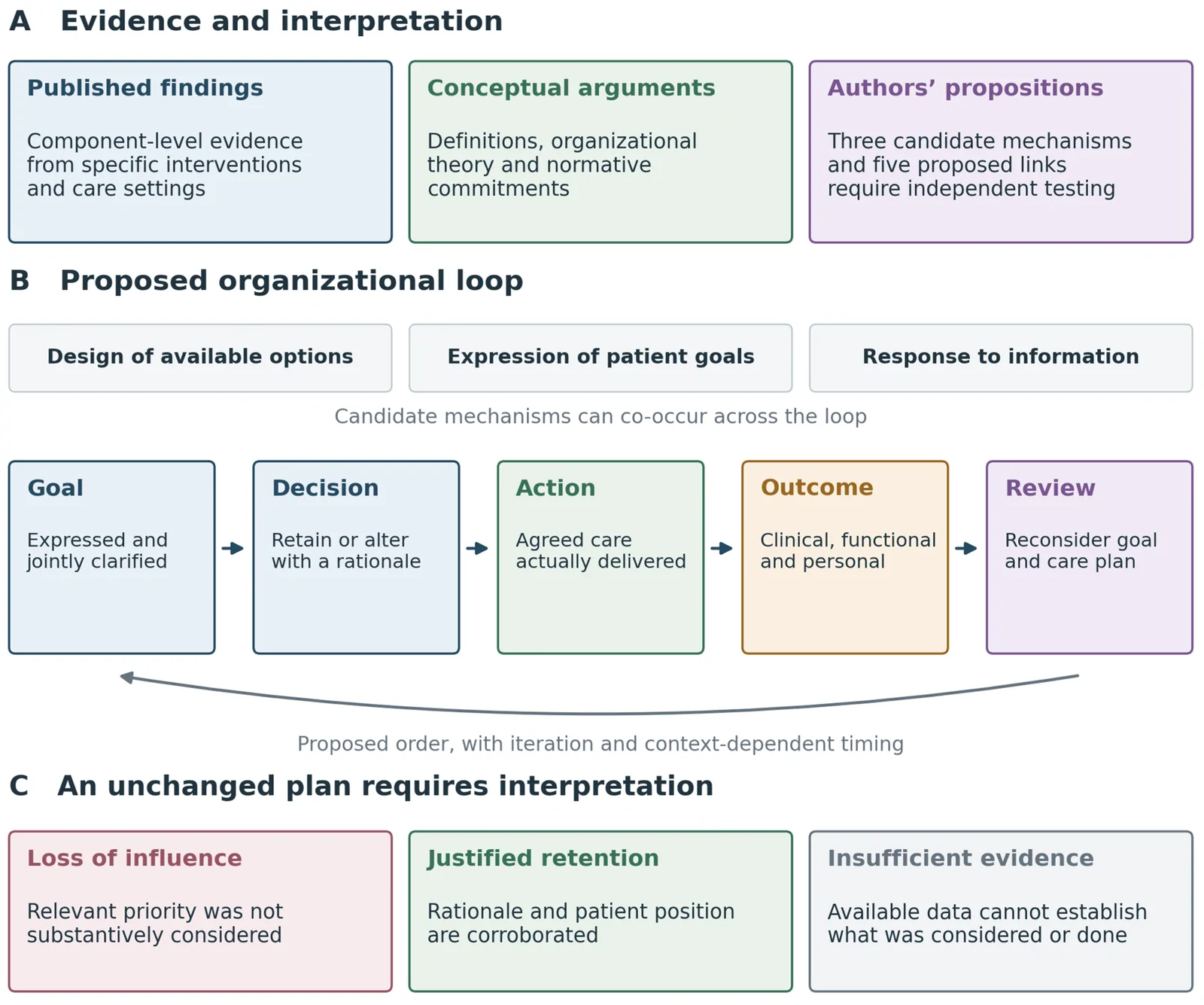
Evidence, the proposed loop, and interpretation of an unchanged care plan.

**Figure 2:**
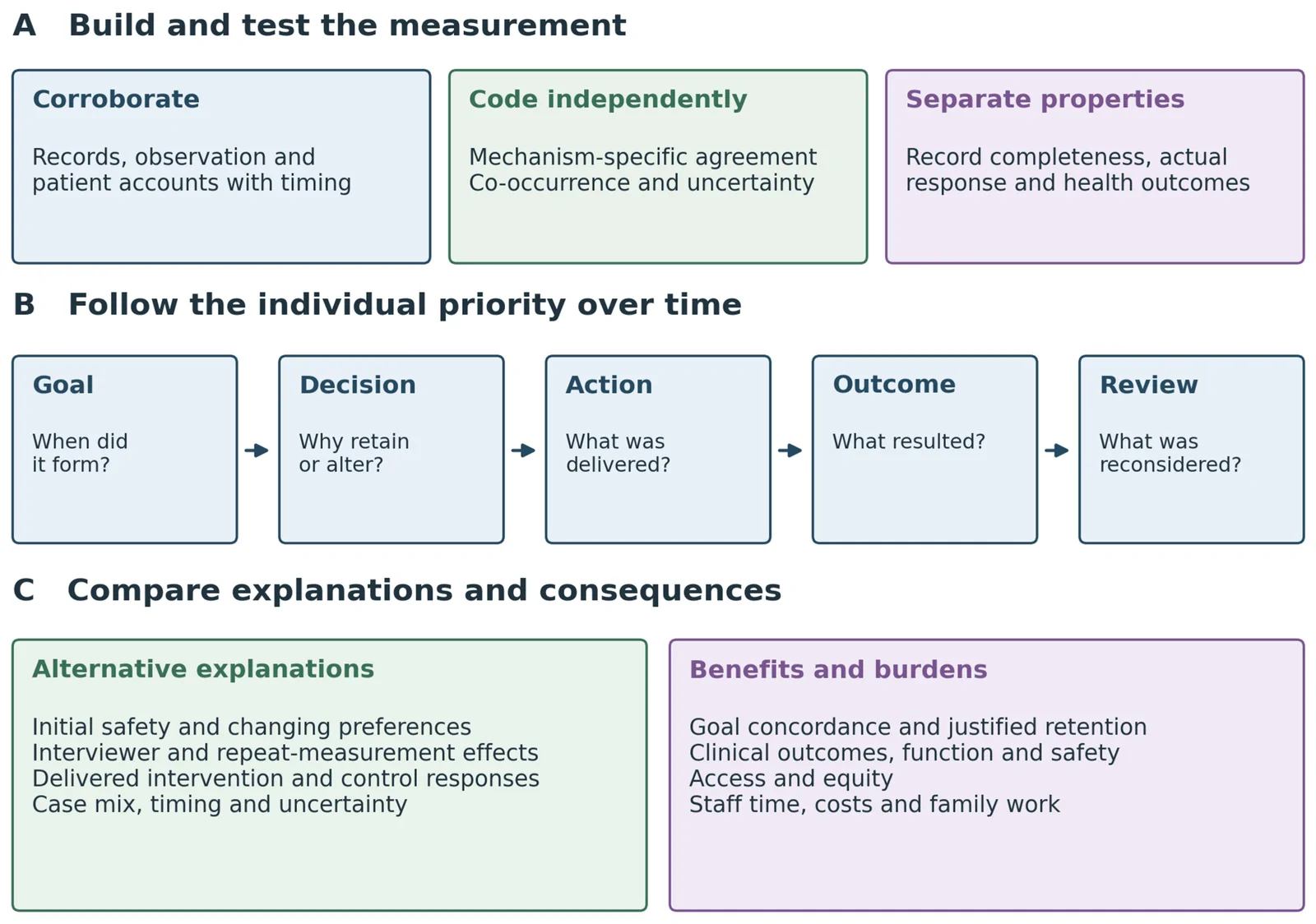
Proposed programme for measurement and empirical testing.

**Table 1:** The evidence base and the limits of transferring conclusions.

Domain	Nature of the Evidence Used	What It Supports	What It Does Not Establish
Organizational implementation and involvement	Organizational studies, qualitative research, and reviews of implementation barriers [10,11,30,31,32]	Policy and participation do not establish enacted influence. Leadership, roles, and resources can facilitate implementation.	Prevalence, causal importance, or completeness of the proposed mechanisms. Related reports are not independent replications.
Voice and relationships of dependency	Qualitative studies across several types of care, including patient safety and intensive care [14,15,16,33]	Anticipated reactions, professional authority, and dependency can constrain the expression of dissent.	How prevalent the mechanism is across all sectors, and whether silence invariably signals a suppressed voice.
Experience and outcome measures	Reviews of PREM and PROM feedback, including randomized trials [12,13,18,19,20,21,22,34]	Effects vary by outcome and intervention. Some feedback interventions improve processes and health outcomes.	That all measurement is ineffective, or that every proposed response component is necessary for benefit.
Goal concordance	Reviews of instruments and studies of seriously ill patients [35,36,37]	Preferences, care delivered, alignment, and outcome attainment can be examined as distinct properties.	A universal instrument or threshold, or transfer of measures from geriatrics and palliative care without adaptation.
Recovery and capability	Conceptual models and studies of quality of life, rehabilitation, and mental health [38,39,40,41]	A meaningful outcome may encompass function, adaptation, and the capacity to live with persisting disease.	A causal effect of organizational personalization, or the applicability of a single recovery model to all conditions.
Value and resource use	A conceptual value framework, reviews, observational work, and a decision-support trial [42,43,44,45,46]	Specific interventions may affect use and costs. Conceptual work defines what should be valued.	Automatic savings, causal effects from associations, or benefit where access worsens or family burden rises.

**Table 2:** Putative mechanisms of organizational decoupling and the data needed to test them.

Candidate Mechanism	Evidence Suggesting a Loss of Influence	Justified Retention or Alternative Explanation	Evidence Needed for Interpretation
Representation and design	A relevant patient contribution is excluded from substantive consideration of options, rules, or resources.	The proposal was considered but was unsuitable or infeasible for stated reasons. Existing options already address the individual priority.	Trace the contribution through deliberation, authority, implementation and individual use. A council or an unchanged pathway alone is insufficient.
Expression and recognition	The patient describes a pre-existing priority or disagreement that was withheld because expression felt unsafe or ineffective.	The initial conversation was already safe. The preference changed later, or the patient freely delegated the decision. Repeated questioning affected the answer.	Compare time-anchored accounts, reasons for change, and observed interaction. Include repeat-measurement controls. A changed answer alone does not prove suppressed voice.
Measurement and response	Relevant information reached the team, but received no consideration or follow-up when a response was indicated.	The information was reviewed and justified retaining the plan. A response occurred outside the formal record, or the observation window was too short.	Link the signal to a decision rationale, any action, and an appropriate review. Verify delivered care separately from record completion.

**Table 3:** Testable propositions of the framework.

No.	Testable Proposition	Design and Alternative Explanations	Finding That Would Weaken the Proposition
P1	Authority and resources for considering relevant individual priorities improve goal concordance beyond a formal policy alone.	Compare organizations and pathways while accounting for case mix, baseline concordance, existing flexibility, and informal authority. Verify what options patients actually needed.	A precise comparison excludes a meaningful advantage of these provisions where adaptation is needed. Good results in pathways requiring no adaptation do not test the claim.
P2	Where expression is constrained, making dissent safer increases recognition of pre-existing priorities in decisions.	Assess initial safety independently. Record when priorities formed and what information changed. Compare protected elicitation with an equally timed repeat interview, balancing interviewer effects and desired delegation.	Despite a verified improvement in safety and reliable measurement, estimates exclude a meaningful improvement in recognition. Stable answers in an already-safe setting are inconclusive.
P3	Feedback with an explicit organizational response improves relevant action or outcomes more than passive reporting.	Use randomized or controlled phased implementation. Verify delivered responses in both groups. Account for observation-related attention, case mix, timing, clustering, and missing data. Separate process and health outcomes.	With a meaningful delivered contrast, confidence intervals exclude a prespecified useful advantage. A non-significant difference with wide intervals or poor delivery remains inconclusive.
P4	A genuinely absent relevant link weakens the association between patient priorities and subsequent care.	Follow priorities using observation, interviews, and delivered care. Distinguish absent, undocumented, and unnecessary links. Address selection of simpler cases and general organizational attention. Assess health outcomes separately.	Reliable observation shows concordant care despite the absence of a supposedly necessary link, or a precise estimate excludes a meaningful association. This challenges necessity in that context.
P5	Pathway complexity and transfers between services increase the risk of losing patient influence.	Compare pathways using equivalent definitions and observation across languages and settings. Account for illness severity, resources, communication support, and data completeness. Repeat comparisons in independent samples.	After these differences are addressed, estimates exclude a meaningful increase or show less loss with greater complexity. Failure to detect a difference in sparse data is inconclusive.
P6	Any economic benefit partly operates through less discordant or low-value care.	Measure costs, care use, the timing of proposed mediators, clinical outcomes, access, and unpaid family work. Use a suitable causal design for mediation claims.	Well-measured savings occur without change in the proposed mediators, weakening that pathway. Savings through restricted access or transferred burden do not establish benefit.

## Data Availability

No original patient-level dataset was analyzed. The analytical bibliography, source-to-claim matrix, illustrative search expressions, and proposed coding rules are provided in the Supplementary Materials. Historical search and screening logs are not available.

## Supplementary Materials

The following supporting information can be downloaded at: https://www.mdpi.com/article/10.3390/healthcare14193136/s1, Table S1: Reported retrieval routes and illustrative search expressions; Figure S1: Account of the analytical reference set; Table S2: Complete Source-to-Claim Matrix; Table S3: From published findings to candidate mechanisms; Table S4: Proposed operational coding rules.
