# Supplementary material for: When Personalization Does Not Change Care: A Critical Narrative Review and an Organizational Decoupling Framework
Source: Healthcare (Basel). 2026 Sep 22;14(19):3136. doi: 10.3390/healthcare14193136 (PMC13665140; doi:10.3390/healthcare14193136)
Supplement: Supplementary file 1 [file healthcare-14-03136-s001.zip › healthcare-4523041-supplementary.pdf]

Supplementary Materials for healthcare-4523041

When Personalization Does Not Change Care: A Critical Narrative Review and an Organizational Decoupling Framework

Table S1 Reported Retrieval Routes and Illustrative Search Expressions

The preparation text reports targeted PubMed/MEDLINE retrieval, Crossref bibliographic checks, reference list reading, forward citation following, and journal websites during July and early August 2026. Exact database histories, search-specific dates, deduplication counts, and record-level exclusion decisions were not retained. Crossref was a metadata resource. No independent duplicate screening or formal risk-of-bias assessment was conducted.

Only three example term combinations were preserved in the preparation text. They are reproduced below as reported examples, without a claim that the complete historical search can be recovered.

(person-cent* OR patient-cent* OR people-cent*) AND (implementation OR organization* OR policy-practice OR decoupl*)

(patient OR service user*) AND (voice OR silence OR speaking up OR difficult patient) AND healthcare*

(PREM* OR PROM* OR patient experience OR goal concordan*) AND (feedback OR action OR quality improvement)

The complete expressions in the following table are illustrative PubMed formulations prepared for this revision. They organize the search themes and are not represented as previously executed queries. No hit counts, search dates, or eligibility flow are assigned to them. The choice of the analytical sources remains purposive.

The selection rationale was conceptual relevance to how priorities are expressed, considered in decisions, and followed through care, including organizational conditions, outcomes, and resources. Conceptual, methodological, and empirical publications could contribute. Successful implementation, positive effects, and limitations were retained where relevant. Molecular stratification or clinical efficacy without an organizational connection fell outside this argument. These are retrospective descriptions of the bibliography, not preserved decisions for individual excluded records.

Theme	Illustrative expression	Purpose and status
**Centredness and concepts**	("patient-centered care"[Title/Abstract] OR "patient-centred care"[Title/Abstract] OR "person-centered care"[Title/Abstract] OR "person-centred care"[Title/Abstract] OR personalization[Title/Abstract] OR personalisation[Title/Abstract]) AND (concept*[Title/Abstract] OR definition*[Title/Abstract] OR framework*[Title/Abstract] OR review[Title/Abstract])	Definitions and relationships among approaches.
**Organization and decoupling**	("patient-centered care"[Title/Abstract] OR "person-centred care"[Title/Abstract] OR personalization[Title/Abstract]) AND (implementation[Title/Abstract] OR organization*[Title/Abstract] OR organisation*[Title/Abstract] OR decoupl*[Title/Abstract] OR "policy-practice"[Title/Abstract])	Organizational theory and healthcare implementation. Adapted from a retained theme, not a recovered query.

**Voice and dependency**	(patient*[Title/Abstract] OR "service user"[Title/Abstract]) AND (voice[Title/Abstract] OR silence[Title/Abstract] OR "speaking up"[Title/Abstract] OR "difficult patient"[Title/Abstract] OR "epistemic injustice"[Title/Abstract]) AND (healthcare[Title/Abstract] OR "health care"[Title/Abstract])	Expression, participation, and relational constraints. Adapted from a retained theme.
**Measurement and response**	("patient reported outcome"[Title/Abstract] OR "patient-reported outcome measures"[Title/Abstract] OR PROMs[Title/Abstract] OR PREMs[Title/Abstract] OR "patient experience"[Title/Abstract]) AND (feedback[Title/Abstract] OR action[Title/Abstract] OR "quality improvement"[Title/Abstract] OR implementation[Title/Abstract])	Effects of measurement and organizational uses. Adapted from a retained theme.
**Shared decisions**	("shared decision making"[Title/Abstract] OR "shared decision-making"[Title/Abstract]) AND (implementation[Title/Abstract] OR barrier*[Title/Abstract] OR facilitator*[Title/Abstract] OR model[Title/Abstract] OR outcome*[Title/Abstract])	Deliberation, implementation, and follow-up.
**Coproduction and collective involvement**	(coproduction[Title/Abstract] OR "co-production"[Title/Abstract] OR "co-design"[Title/Abstract] OR "patient participation"[Title/Abstract] OR "patient involvement"[Title/Abstract]) AND (healthcare[Title/Abstract] OR "health care"[Title/Abstract]) AND (service*[Title/Abstract] OR organization*[Title/Abstract] OR organisation*[Title/Abstract] OR quality[Title/Abstract])	Participation in service production and design.
**Goals and concordance**	("goal concordance"[Title/Abstract] OR "goal-concordant care"[Title/Abstract] OR "patient priorities"[Title/Abstract]) AND (measure*[Title/Abstract] OR decision*[Title/Abstract] OR care[Title/Abstract])	Goal measurement and alignment of care.
**Recovery and capability**	("personal recovery"[Title/Abstract] OR "health capability"[Title/Abstract] OR "capabilities approach"[Title/Abstract] OR suffering[Title/Abstract] OR "biopsychosocial model"[Title/Abstract]) AND (health*[Title/Abstract] OR care[Title/Abstract] OR illness[Title/Abstract])	Meaningful outcomes and real opportunities to act.
**Value and resource use**	("patient-centered care"[Title/Abstract] OR "patient-centred care"[Title/Abstract] OR "shared decision making"[Title/Abstract] OR "self-management support"[Title/Abstract]) AND (cost*[Title/Abstract] OR utilization[Title/Abstract] OR utilisation[Title/Abstract] OR "resource use"[Title/Abstract] OR value[Title/Abstract])	Economic hypotheses and context-specific findings.

Foundational organizational and philosophical sources were also located through citation following and journal websites. PubMed formulations alone do not cover those literatures. Reconstructing an exact historical strategy would require records that are unavailable. The present table improves transparency about the scope of reading without claiming reproducible selection.

Figure S1 Account of the Analytical Reference Set

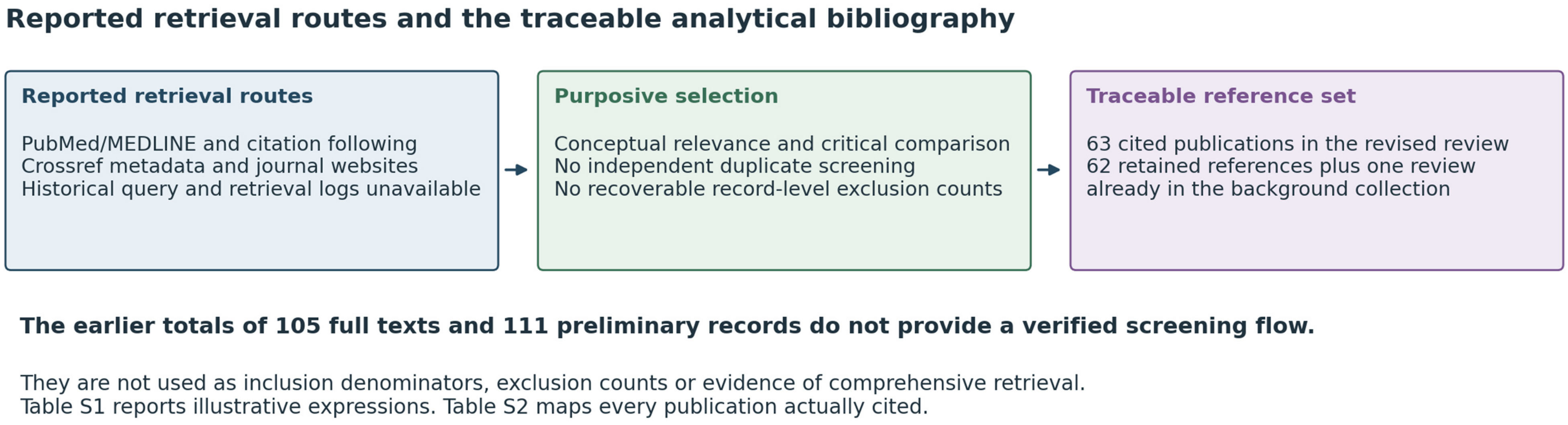

This figure reports routes and a verifiable final bibliography. It is not a PRISMA diagram, and no historical record counts or reasons for individual exclusions have been reconstructed. The previously stated totals of 105 full texts and 111 preliminary records are not used as screened-corpus denominators. The revised analysis maps 63 cited sources, including one Cochrane review already in the background reading collection.

Table S2 Complete Source-to-Claim Matrix

All 63 publications cited in the revised manuscript are listed in citation order. This is a retrospective map of their contribution to the present argument, not a recovered extraction or exclusion log. Design labels distinguish conceptual, methodological, and empirical contributions. Support for a component does not validate the integrated framework. Related Veterans Health Administration reports and overlapping evidence syntheses are not independent replications.

Publication	Design and Role in the Argument	Limits of Inference
**1. Mead, N.; Bower, P. Patient-centredness: a conceptual framework and review of the empirical literature. Social Science & Medicine 2000, 51, 1087–1110. https://doi.org/10.1016/S0277-9536(00)00098-8**	Conceptual framework and literature review. Patient-centredness includes a biopsychosocial view, the patient as a person, shared power, alliance, and the clinician as a person. Supports terminology and the relational argument.	Conceptual and heterogeneous empirical material. Does not establish the three decouplings or their prevalence.

**2. Kitson, A.; Marshall, A.; Bassett, K.; Zeitz, K. What are the core elements of patient-centred care? A narrative review and synthesis of the literature from health policy, medicine and nursing. Journal of Advanced Nursing 2013, 69, 4–15. https://doi.org/10.1111/j.1365-2648.2012.06064.x**	Narrative review and synthesis. Core elements across policy, medicine, and nursing. Supports attention to participation, relationships, and the context of care.	Definitions and disciplinary emphases vary. Does not demonstrate failure of personalization as a whole.
**3. Santana, M.J.; Manalili, K.; Jolley, R.J.; Zelinsky, S.; Quan, H.; Lu, M. How to practice person-centred care: A conceptual framework. Health Expectations 2018, 21, 429–440. https://doi.org/10.1111/hex.12640**	Conceptual framework for person-centred care. Links structures, processes, and outcomes. Establishes that organizational conditions already form part of person-centred care models.	A conceptual framework, not a causal comparison of the proposed mechanisms. Limits claims of novelty.
**4. Scholl, I.; Zill, J.M.; Härter, M.; Dirmaier, J. An Integrative Model of Patient-Centeredness – A Systematic Review and Concept Analysis. PLoS ONE 2014, 9, e107828. https://doi.org/10.1371/journal.pone.0107828**	Systematic review and concept analysis. Integrates dimensions of patient-centredness. Informs conceptual distinctions and comparison with existing multidimensional approaches.	Conceptual integration does not validate a sequence of organizational actions or a quality indicator.
**5. Feldthusen, C.; Forsgren, E.; Wallström, S.; Andersson, V.; Löfqvist, N.; Sawatzky, R.; Öhlén, J.; J. Ung, E. Centredness in health care: A systematic overview of reviews. Health Expectations 2022, 25, 885–901. https://doi.org/10.1111/hex.13461**	Systematic overview of reviews. Examines multiple forms of centredness. Supports recognition of overlap and terminological heterogeneity.	Reviews may share primary studies. Centredness terms and outcome definitions are not interchangeable.
**6. Håkansson Eklund, J.; Holmström, I.K.; Kumlin, T.; Kaminsky, E.; Skoglund, K.; Höglander, J.; Sundler, A.J.; Condén, E.; Summer Meranius, M. "Same same or different?" A review of reviews of person-centered and patient-centered care. Patient Education and Counseling 2019, 102, 3–11. https://doi.org/10.1016/j.pec.2018.08.029**	Review of reviews. Compares patient-centred and person-centred care, including the distinction between functional and meaningful life goals.	The distinction is an interpretation of selected reviews. It is not a universal taxonomy of all uses of either term.
**7. Berwick, D.M. What 'Patient-Centered' Should Mean: Confessions Of An Extremist. Health Affairs 2009, 28, w555–w565. https://doi.org/10.1377/hlthaff.28.4.w555**	Normative essay. Argues for patient influence and attention to individual values. Supports the ethical premise of the review.	A normative position, not empirical evidence of a clinical or organizational effect.
**8. Meyer, J.W.; Rowan, B. Institutionalized Organizations: Formal Structure as Myth and Ceremony. American Journal of Sociology 1977, 83, 340–363. https://doi.org/10.1086/226550**	Foundational organizational theory. Explains how formal organizational structures may become detached from everyday activities. Supplies the policy–practice lens.	General organizational theory. No healthcare-specific estimate or direct validation of this personalization framework.
**9. Bromley, P.; Powell, W.W. From Smoke and Mirrors to Walking the Talk: Decoupling in the Contemporary World. Academy of Management Annals 2012, 6, 483–530. https://doi.org/10.1080/19416520.2012.684462**	Theoretical review of organizational decoupling. Distinguishes policy–practice from means–ends decoupling. Supports examining implemented procedures whose intended benefit remains uncertain.	The three candidate mechanisms are an application by the present authors, not categories established by this source.
**10. Waddell, A.; Lennox, A.; Spassova, G.; Bragge, P. Barriers and facilitators to shared decision-making in hospitals from policy to practice: a systematic review. Implementation Science 2021, 16, 74. https://doi.org/10.1186/s13012-021-01142-y**	Systematic review of hospital implementation. Identifies barriers and facilitators to shared decision making across policy and practice. Supports attention to time, teams, and organizational priorities.	Concerns implementation of shared decision making. It does not establish the effectiveness of the proposed five-link loop.

**11. Westerink, H.J.; Oirbans, T.; Garvelink, M.M.; van Uden-Kraan, C.F.; Zouitni, O.; Bart, H.A.; van der Wees, P.J.; van der Nat, P.B. Barriers and facilitators of meaningful patient participation at the collective level in healthcare organizations: A systematic review. Health Policy 2023, 138, 104946. https://doi.org/10.1016/j.healthpol.2023.104946**	Systematic review of collective participation. Identifies barriers and facilitators to meaningful participation in healthcare organizations. Supports separating invitation from influence over service design.	Collective participation cannot establish concordance with an individual patient's goal. Outcomes and contexts are heterogeneous.
**12. Gleeson, H.; Calderon, A.; Swami, V.; Deighton, J.; Wolpert, M.; Edbrooke-Childs, J. Systematic review of approaches to using patient experience data for quality improvement in healthcare settings. BMJ Open 2016, 6, e011907. https://doi.org/10.1136/bmjopen-2016-011907**	Systematic review of patient experience data use. Describes quality-improvement responses and limited evaluation of resulting changes. Supports distinguishing collection from organizational action.	Limited evaluation is not proof that patient experience data are ineffective. Reported changes differ in scope.
**13. Jamieson Gilmore, K.; Corazza, I.; Coletta, L.; Allin, S. The uses of Patient Reported Experience Measures in health systems: A systematic narrative review. Health Policy 2023, 128, 1–10. https://doi.org/10.1016/j.healthpol.2022.07.008**	Systematic narrative review of PREM use. Maps uses across system levels and identifies limited evidence linking organizational- or system-level initiatives to evaluated local improvements.	The gap concerns the evidence and implementation chain. It does not establish that all PREMs lack benefit.
**14. Frosch, D.L.; May, S.G.; Rendle, K.A.; Tietbohl, C.; Elwyn, G. Authoritarian Physicians And Patients' Fear Of Being Labeled 'Difficult' Among Key Obstacles To Shared Decision Making. Health Affairs 2012, 31, 1030–1038. https://doi.org/10.1377/hlthaff.2011.0576**	Qualitative focus group study. Participants describe deference and fear of being labelled difficult. Supports a possible relational constraint on expression.	Selected participants and reported experiences. Cannot estimate a universal prevalence of suppressed preferences.
**15. Entwistle, V.A.; McCaughan, D.; Watt, I.S.; Birks, Y.; Hall, J.; Peat, M.; Williams, B.; Wright, J. Speaking up about safety concerns: multi-setting qualitative study of patients' views and experiences. BMJ Quality & Safety 2010, 19, e33–e33. https://doi.org/10.1136/qshc.2009.039743**	Qualitative study across care settings. Examines how relationships, the nature of a concern, and expected responses affect speaking up about safety.	Safety concerns are not identical to treatment preferences. Transfer to other kinds of patient contribution requires testing.
**16. Bell, S.K.; Roche, S.D.; Mueller, A.; Dente, E.; O'Reilly, K.; Sarnoff Lee, B.; Sands, K.; Talmor, D.; Brown, S.M. Speaking up about care concerns in the ICU: patient and family experiences, attitudes and perceived barriers. BMJ Quality & Safety 2018, 27, 928–936. https://doi.org/10.1136/bmjqs-2017-007525**	Patient and family study in intensive care. Reports perceived barriers to speaking up, including reluctance to trouble staff. Illuminates dependency under acute vulnerability.	Specific ICU context and mixed patient/family perspectives. Frequencies cannot be transferred to other specialties or countries.
**17. Doyle, C.; Lennox, L.; Bell, D. A systematic review of evidence on the links between patient experience and clinical safety and effectiveness. BMJ Open 2013, 3, e001570. https://doi.org/10.1136/bmjopen-2012-001570**	Systematic review. Reports associations between patient experience, safety, and effectiveness. Counters dismissal of experience as irrelevant to quality.	Associations across heterogeneous studies do not establish causation or validate satisfaction as a proxy for goal concordance.
**18. Boyce, M.B.; Browne, J.P. Does providing feedback on patient-reported outcomes to healthcare professionals result in better outcomes for patients? A systematic review. Quality of Life Research 2013, 22, 2265–2278. https://doi.org/10.1007/s11136-013-0390-0**	Systematic review of PROM feedback. Reports inconsistent effects of providing outcome feedback to professionals. Supports attention to intervention design and outcome differences.	An earlier evidence base with heterogeneous interventions. Later positive evidence qualifies a general claim of ineffectiveness.
**19. Wittich, L.; Tsatsaronis, C.; Kuklinski, D.; Schöner, L.; Steinbeck, V.; Busse, R.; Rombey, T. Patient-Reported Outcome Measures as an Intervention: A Comprehensive Overview of Systematic Reviews on the Effects of Feedback. Value in Health 2024, 27, 1436–1453. https://doi.org/10.1016/j.jval.2024.05.013**	Overview of 40 systematic reviews. Finds clearer effects of PROM feedback on processes than on patient health outcomes, with generally low review quality. Informs the conditional response hypothesis.	Overlapping primary studies and varied review quality limit independent replication and comparisons across intervention types.

**20. Bonsel, J.M.; Itiola, A.J.; Huberts, A.S.; Bonsel, G.J.; Penton, H. The use of patient-reported outcome measures to improve patient-related outcomes – a systematic review. Health and Quality of Life Outcomes 2024, 22, 101. https://doi.org/10.1186/s12955-024-02312-4**	Systematic review of PROM interventions. Reports differing results across feedback, monitoring, screening, and benchmarking uses. Suggests that the use of data within care matters.	Between-study differences cannot isolate response arrangements as the cause of benefit. Some studies report benefits from measurement alone.
**21. Graupner, C.; Kimman, M.L.; Mul, S.; Slok, A.H.M.; Claessens, D.; Kleijnen, J.; Dirksen, C.D.; Breukink, S.O. Patient outcomes, patient experiences and process indicators associated with the routine use of patient-reported outcome measures (PROMs) in cancer care: a systematic review. Supportive Care in Cancer 2021, 29, 573–593. https://doi.org/10.1007/s00520-020-05695-4**	Systematic review in cancer care. Describes predominantly positive findings for routine PROM use and feedback in cancer care. Provides a counterargument to blanket criticism of measurement.	Many included studies were underpowered. Findings are context-bound and do not establish every component of the present model.
**22. Gibbons, C.; Porter, I.; Gonçalves-Bradley, D.C.; Stoilov, S.; Ricci-Cabello, I.; Tsangaris, E.; Gangannagaripalli, J.; Davey, A.; Gibbons, E.J.; Kotzeva, A.; Evans, J.; van der Wees, P.J.; Kontopantelis, E.; Greenhalgh, J.; Bower, P.; Alonso, J.; Valderas, J.M. Routine provision of feedback from patient-reported outcome measurements to healthcare providers and patients in clinical practice. Cochrane Database of Systematic Reviews 2021, 10, CD011589. https://doi.org/10.1002/14651858.CD011589.pub2**	Cochrane review of 116 randomized trials. PROM feedback probably produces a small quality of life benefit and improves some processes. Provides a substantive counterexample to a blanket claim of measurement failure.	All trials were from high-income countries. Certainty and effects differ by outcome, with risk of performance and detection bias. Does not prove all proposed response components are necessary.
**23. Tieu, M.; Mudd, A.; Conroy, T.; Pinero de Plaza, A.; Kitson, A. The trouble with personhood and person-centred care. Nursing Philosophy 2022, 23, e12381. https://doi.org/10.1111/nup.12381**	Philosophical analysis. Examines conceptual problems in personhood and person-centred care. Supports the need to state what the present working definition includes.	Conceptual criticism does not demonstrate a clinical or organizational failure rate.
**24. Latif, A.; Gulzar, N. The Fallacy of Person-Centred Care: Deconstructing the Discourse to Reimagine Practice. Nursing Philosophy 2025, 26, e70043. https://doi.org/10.1111/nup.70043**	Critical philosophical discussion. Questions the discourse and enactment of person-centred care. Provides a critical perspective alongside implementation evidence.	An argumentative contribution, not empirical proof that person-centred care is ineffective or absent.
**25. Summer Meranius, M.; Holmström, I.K.; Håkansson, J.; Breitholtz, A.; Moniri, F.; Skogevall, S.; Skoglund, K.; Rasoal, D. Paradoxes of person-centred care: A discussion paper. Nursing Open 2020, 7, 1321–1329. https://doi.org/10.1002/nop2.520**	Discussion paper. Examines paradoxes of person-centred care. Supports retaining tensions and competing demands in the argument.	Conceptual analysis. Does not validate the proposed taxonomy or sequence.
**26. Han, C.J. A Concept Analysis of Personalized Health Care in Nursing. Nursing Forum 2016, 51, 32–39. https://doi.org/10.1111/nuf.12117**	Concept analysis in nursing. Shows that personalized healthcare can include individualized care, collaboration, personal vulnerabilities, and molecular information.	Its definition is broader in some respects than this review's organizational focus. It does not justify treating personalized care as only procedures.
**27. Greenhalgh, T.; Thorne, S.; Malterud, K. Time to challenge the spurious hierarchy of systematic over narrative reviews? European Journal of Clinical Investigation 2018, 48, e12931. https://doi.org/10.1111/eci.12931**	Review methodology argument. Challenges a simple hierarchy placing all systematic reviews above narrative reviews. Supports matching review design to the question.	Does not excuse incomplete reporting or remove the need to recognize selection bias.

**28. Grant, M.J.; Booth, A. A typology of reviews: an analysis of 14 review types and associated methodologies. Health Information & Libraries Journal 2009, 26, 91–108. https://doi.org/10.1111/j.1471-1842.2009.00848.x**	Typology of review methods. Distinguishes review types and their methodological expectations. Informs the explanation of the chosen design and alternatives.	A design reference, not a source of substantive evidence on personalization.
**29. Jabareen, Y. Building a Conceptual Framework: Philosophy, Definitions, and Procedure. International Journal of Qualitative Methods 2009, 8, 49–62. https://doi.org/10.1177/160940690900800406**	Methodological paper on conceptual frameworks. Provides an approach to constructing and examining a conceptual framework across heterogeneous sources.	Citing the method does not establish that every described procedure was implemented in this review.
**30. Bokhour, B.G.; Fix, G.M.; Mueller, N.M.; Barker, A.M.; Lavela, S.L.; Hill, J.N.; Solomon, J.L.; VanDeusen Lukas, C. How can healthcare organizations implement patient-centered care? Examining a large-scale cultural transformation. BMC Health Services Research 2018, 18, 168. https://doi.org/10.1186/s12913-018-2949-5**	Qualitative US Veterans Affairs study. Interviews with 108 employees at 4 centres describe 7 domains of cultural transformation and difficulties aligning roles and rules.	Selected leading centres and staff perspectives. Related to the programme examined in reference 31, so not an independent replication of all findings.
**31. Fix, G.M.; VanDeusen Lukas, C.; Bolton, R.E.; Hill, J.N.; Mueller, N.; LaVela, S.L.; Bokhour, B.G. Patient-centred care is a way of doing things: How healthcare employees conceptualize patient-centred care. Health Expectations 2018, 21, 300–307. https://doi.org/10.1111/hex.12615**	Qualitative US Veterans Health Administration study. Interviews with 107 employees at 4 centres identify varied interpretations, from established constructs to broader culture and traditional care.	Conceptualizations by staff do not establish patient-experienced concordance. Related programme and settings to reference 30.
**32. Baethge, C.; Goldbeck-Wood, S.; Mertens, S. SANRA—a scale for the quality assessment of narrative review articles. Research Integrity and Peer Review 2019, 4, 5. https://doi.org/10.1186/s41073-019-0064-8**	Development and evaluation of SANRA. Provides six domains for assessing narrative review reporting and reasoning. Supports editorial checking.	SANRA does not replace study-level risk-of-bias assessment, independent review, or a search record.
**33. Makoul, G.; Clayman, M.L. An integrative model of shared decision making in medical encounters. Patient Education and Counseling 2006, 60, 301–312. https://doi.org/10.1016/j.pec.2005.06.010**	Integrative model of shared decision making. Describes essential elements of deliberation and decision processes. Supplies a comparator and includes arrangements beyond simply offering options.	The present framework should not claim that shared decision making lacks follow-up or organizational relevance.
**34. Elwyn, G.; Frosch, D.; Thomson, R.; Joseph-Williams, N.; Lloyd, A.; Kinnersley, P.; Cording, E.; Tomson, D.; Dodd, C.; Rollnick, S.; Edwards, A.; Barry, M. Shared Decision Making: A Model for Clinical Practice. Journal of General Internal Medicine 2012, 27, 1361–1367. https://doi.org/10.1007/s11606-012-2077-6**	Clinical model of shared decision making. Describes choice, option, and decision talk. Supports collaborative deliberation and review of decisions as appropriate.	A practice model, not validation of the organizational sequence proposed here.
**35. Batalden, M.; Batalden, P.; Margolis, P.; Seid, M.; Armstrong, G.; Opipari-Arrigan, L.; Hartung, H. Coproduction of healthcare service. BMJ Quality & Safety 2016, 25, 509–517. https://doi.org/10.1136/bmjqs-2015-004315**	Conceptual paper on healthcare coproduction. Treats services as produced through interactions between patients and professionals. Connects individual participation to care delivery.	Coproduction is broader than collective co-design. It cannot be reduced to a patient council or a single treatment choice.
**36. Elwyn, G.; Nelson, E.; Hager, A.; Price, A. Coproduction: when users define quality. BMJ Quality & Safety 2020, 29, 711–716. https://doi.org/10.1136/bmjqs-2019-009830**	Conceptual discussion of coproduction and quality. Examines the contribution of service users to defining quality. Informs the link between what matters and evaluation of care.	Does not establish that group-defined quality criteria represent each individual's priorities.

**37. Ocloo, J.; Matthews, R. From tokenism to empowerment: progressing patient and public involvement in healthcare improvement. BMJ Quality & Safety 2016, 25, 626–632. https://doi.org/10.1136/bmjqs-2015-004839**	Critical narrative review of public involvement. Examines tokenism, unequal power, and participation in designing or improving services. Supports distinguishing representation from substantive influence.	Collective involvement and individual clinical decisions are different units. No independent validation of the three-category model.
**38. Joseph-Williams, N.; Elwyn, G.; Edwards, A. Knowledge is not power for patients: A systematic review and thematic synthesis of patient-reported barriers and facilitators to shared decision making. Patient Education and Counseling 2014, 94, 291–309. https://doi.org/10.1016/j.pec.2013.10.031**	Systematic review and thematic synthesis. Identifies patient-reported barriers involving knowledge, power, and the organization of care. Supports the possibility of constrained participation.	Shared decision-making barriers do not establish suppressed voice in an individual case. Independent, time-sensitive corroboration is needed.
**39. Luxford, K.; Safran, D.G.; Delbanco, T. Promoting patient-centered care: a qualitative study of facilitators and barriers in healthcare organizations with a reputation for improving the patient experience. International Journal for Quality in Health Care 2011, 23, 510–515. https://doi.org/10.1093/intqhc/mzr024**	Qualitative study in eight US organizations. Interviews with 40 senior staff and patient representatives describe leadership, engagement, resources, and culture as implementation facilitators.	Organizations were selected for a reputation for patient-centred care. Findings do not isolate causation or guarantee transfer to less-resourced settings.
**40. Bull, C.; Byrnes, J.; Hettiarachchi, R.; Downes, M. A systematic review of the validity and reliability of patient-reported experience measures. Health Services Research 2019, 54, 1023–1035. https://doi.org/10.1111/1475-6773.13187**	Systematic review of PREM measurement properties. Shows variation in the examination of validity and reliability. Supports careful selection of measures and separation of construct from instrument.	Measurement properties do not determine whether organizations respond to results or validate the proposed mechanisms.
**41. Chua, I.S.; Berler, A.; Ninteau, K.; Bain, P.A.; Fromme, E.K.; Edelen, M.; Pusic, A.L.; Ritchie, C.S.; Bates, D.W. Patient-Centered Measures of Goal Concordance in Geriatrics and Palliative Care: A Scoping Review. JAMA Network Open 2025, 8, e2530370. https://doi.org/10.1001/jamanetworkopen.2025.30370**	Scoping review in geriatrics and palliative care. Identifies 44 unique measures within 63 studies and four conceptual components of goal-concordance measurement.	Preference alignment and outcome attainment are different. No universal standard or cross-specialty threshold is established.
**42. Ernecoff, N.C.; Wessell, K.L.; Bennett, A.V.; Hanson, L.C. Measuring Goal-Concordant Care in Palliative Care Research. Journal of Pain and Symptom Management 2021, 62, e305–e314. https://doi.org/10.1016/j.jpainsymman.2021.02.030**	Methodological review in palliative care. Examines strategies and limitations for measuring goal-concordant care, including timing and proxy perspectives.	Methods require adaptation outside the source context. Records and retrospective accounts are imperfect measures of influence.
**43. Modes, M.E.; Heckbert, S.R.; Engelberg, R.A.; Nielsen, E.L.; Curtis, J.R.; Kross, E.K. Patient-Reported Receipt of Goal-Concordant Care Among Seriously Ill Outpatients—Prevalence and Associated Factors. Journal of Pain and Symptom Management 2020, 60, 765–773. https://doi.org/10.1016/j.jpainsymman.2020.04.026**	Secondary analysis of trial baseline surveys. Among 405 seriously ill US outpatients, 58% reported concordance, 17% discordance, and 25% uncertainty about the focus of care.	An observational baseline analysis despite trial provenance. Measures reported concordance, not the prevalence or causes of the three decouplings.
**44. Engel, G.L. The Need for a New Medical Model: A Challenge for Biomedicine. Science 1977, 196, 129–136. https://doi.org/10.1126/science.847460**	Foundational biopsychosocial argument. Locates illness within biological, psychological, and social dimensions. Supports a broader interpretation of outcomes.	Conceptual model, not evidence that the organizational loop improves recovery.

**45. Leamy, M.; Bird, V.; Boutillier, C.L.; Williams, J.; Slade, M. Conceptual framework for personal recovery in mental health: systematic review and narrative synthesis. British Journal of Psychiatry 2011, 199, 445–452. https://doi.org/10.1192/bjp.bp.110.083733**	Systematic review and narrative synthesis in mental health. Develops a personal recovery framework involving connectedness, hope, identity, meaning, and empowerment.	Mental health framework. Its application to surgery or other specialties requires conceptual and empirical evaluation.
**46. Wilson, I.B.; Cleary, P.D. Linking Clinical Variables With Health-Related Quality of Life: A Conceptual Model of Patient Outcomes. JAMA 1995, 273, 59–65. https://doi.org/10.1001/jama.1995.03520250075037**	Conceptual model of health-related quality of life. Relates biological variables, symptoms, function, perceived health, and overall quality of life. Supports distinct outcome domains.	The model does not make an individual's goals equivalent to clinical outcome measures or establish the present causal chain.
**47. Wade, D.T.; Halligan, P.W. The biopsychosocial model of illness: a model whose time has come. Clinical Rehabilitation 2017, 31, 995–1004. https://doi.org/10.1177/0269215517709890**	Biopsychosocial conceptual review. Discusses illness, rehabilitation, and the relationship between clinical and broader life outcomes.	Supports interpretation of outcome, not an effect estimate for organizational personalization.
**48. Porter, M.E. What Is Value in Health Care?. New England Journal of Medicine 2010, 363, 2477–2481. https://doi.org/10.1056/NEJMp1011024**	Conceptual perspective on value. Relates health outcomes to the costs of a care cycle. Informs what an economic evaluation would need to consider.	A value framework, not evidence of automatic savings or a complete measure of individual goal concordance.
**49. Rathert, C.; Wyrwich, M.D.; Boren, S.A. Patient-Centered Care and Outcomes: A Systematic Review of the Literature. Medical Care Research and Review 2013, 70, 351–379. https://doi.org/10.1177/1077558712465774**	Systematic review of patient-centred care and outcomes. Finds more consistent positive relationships for satisfaction and self-management than for clinical outcomes.	Intervention and outcome heterogeneity. Cannot establish an effect of the proposed decoupling framework.
**50. Bertakis, K.D.; Azari, R. Patient-Centered Care is Associated with Decreased Health Care Utilization. The Journal of the American Board of Family Medicine 2011, 24, 229–239. https://doi.org/10.3122/jabfm.2011.03.100170**	Observational primary-care study. Associates a patient-centred consultation style with lower subsequent healthcare use and charges.	Potential confounding and setting-specific costs. Causal savings cannot be inferred.
**51. Veroff, D.; Marr, A.; Wennberg, D.E. Enhanced Support For Shared Decision Making Reduced Costs Of Care For Patients With Preference-Sensitive Conditions. Health Affairs 2013, 32, 285–293. https://doi.org/10.1377/hlthaff.2011.0941**	One-year US randomized investigation. Enhanced remote decision support was associated with lower medical costs, admissions, and preference-sensitive surgery than usual support.	A specific support intervention, population, and payment context. Not a test of the proposed organizational loop.
**52. Panagioti, M.; Richardson, G.; Small, N.; Murray, E.; Rogers, A.; Kennedy, A.; Newman, S.; Bower, P. Self-management support interventions to reduce health care utilisation without compromising outcomes: a systematic review and meta-analysis. BMC Health Services Research 2014, 14, 356. https://doi.org/10.1186/1472-6963-14-356**	Systematic review and meta-analysis. Examines self-management support and service use without compromising outcomes. Supports the possibility of context-specific economic benefit.	Varied conditions and interventions. Benefits cannot be assumed for personalization in general, and burden transfer needs separate assessment.
**53. Schuttner, L.; Hockett Sherlock, S.; Simons, C.E.; Johnson, N.L.; Wirtz, E.; Ralston, J.D.; Rosland, A.M.; Nelson, K.; Sayre, G. My Goals Are Not Their Goals: Barriers and Facilitators to Delivery of Patient-Centered Care for Patients with Multimorbidity. Journal of General Internal Medicine 2022, 37, 4189–4196. https://doi.org/10.1007/s11606-022-07533-1**	Qualitative study of multimorbidity care. Clinicians describe goal differences, time limits, coordination, and policy barriers. Informs the hypothesis about complex pathways.	Reports clinicians' perspectives. Does not demonstrate that decoupling is more frequent in every complex pathway.

**54. Carel, H.; Kidd, I.J. Epistemic injustice in healthcare: a philosophial analysis. Medicine, Health Care and Philosophy 2014, 17, 529–540. https://doi.org/10.1007/s11019-014-9560-2**	Philosophical analysis of epistemic injustice. Explains how patient testimony can be discounted and experiences can lack a recognized interpretive vocabulary. Supplies a relational explanation.	The argument is conceptual. It does not show that silence, a changed interview response, or distress proves a suppressed preference.
**55. Fisher, K.A.; Gallagher, T.H.; Smith, K.M.; Zhou, Y.; Crawford, S.; Amroze, A.; Mazor, K.M. Communicating with patients about breakdowns in care: a national randomised vignette-based survey. BMJ Quality & Safety 2020, 29, 313–319. https://doi.org/10.1136/bmjqs-2019-009712**	Randomized vignette-based survey. Tests how invitations to report breakdowns affect stated willingness to speak up.	Responses to scenarios are not actual disclosure, changed treatment, or improved outcomes.
**56. Entwistle, V.A.; Carter, S.M.; Cribb, A.; McCaffery, K. Supporting Patient Autonomy: The Importance of Clinician-patient Relationships. Journal of General Internal Medicine 2010, 25, 741–745. https://doi.org/10.1007/s11606-010-1292-2**	Conceptual analysis of relational autonomy. Explains how supportive clinical relationships can enable autonomy. Supports chosen delegation and assistance with decisions.	Autonomy is not measured by maximum independent choice. The source does not validate protected interviewing as an intervention.
**57. Sitzia, J.; Wood, N. Patient satisfaction: A review of issues and concepts. Social Science & Medicine 1997, 45, 1829–1843. https://doi.org/10.1016/S0277-9536(97)00128-7**	Review of satisfaction concepts and measurement. Describes how expectations and contextual influences shape satisfaction. Supports separation from technical quality and goal concordance.	Conceptual heterogeneity and an older literature. Does not render satisfaction measures inherently invalid.
**58. Fenton, J.J.; Jerant, A.F.; Bertakis, K.D.; Franks, P. The Cost of Satisfaction: A National Study of Patient Satisfaction, Health Care Utilization, Expenditures, and Mortality. Archives of Internal Medicine 2012, 172, 405–411. https://doi.org/10.1001/archinternmed.2011.1662**	Prospective US observational cohort. Higher satisfaction was associated with greater inpatient use, costs, and mortality. Limits satisfaction as an independent proxy for value.	Observational associations do not establish that satisfaction caused those outcomes. Residual confounding and reverse causation remain possible.
**59. Huber, M.; Knottnerus, J.A.; Green, L.; Horst, H.v.d.; Jadad, A.R.; Kromhout, D.; Leonard, B.; Lorig, K.; Loureiro, M.I.; Meer, J.W.M.v.d.; Schnabel, P.; Smith, R.; Weel, C.v.; Smid, H. How should we define health?. BMJ 2011, 343, d4163–d4163. https://doi.org/10.1136/bmj.d4163**	Conceptual discussion of health. Proposes attention to adaptation and self-management. Supports meaningful outcomes despite persistent disease.	A proposed conception of health, not proof of a clinical benefit of personalization.
**60. Entwistle, V.A.; Watt, I.S. Treating Patients as Persons: A Capabilities Approach to Support Delivery of Person-Centered Care. The American Journal of Bioethics 2013, 13, 29–39. https://doi.org/10.1080/15265161.2013.802060**	Capabilities approach to person-centred care. Emphasizes real opportunities to act and live as a person. Supports interpreting agency beyond formal choice.	Normative and conceptual analysis. Does not establish a universal patient-reported outcome or operational threshold.
**61. Prah Ruger, J. Health Capability: Conceptualization and Operationalization. American Journal of Public Health 2010, 100, 41–49. https://doi.org/10.2105/AJPH.2008.143651**	Conceptualization of health capability. Links agency and opportunities for health. Broadens the interpretation of participation and meaningful outcomes.	Its construct is not equivalent to goal concordance or the proposed organizational mechanisms.
**62. Cassel, E.J. The Nature of Suffering and the Goals of Medicine. New England Journal of Medicine 1982, 306, 639–645. https://doi.org/10.1056/NEJM198203183061104**	Clinical and philosophical account of suffering. Explains why suffering concerns the person and cannot be exhausted by symptoms. Supports distinguishing treatment from its personal meaning.	Conceptual argument rather than evidence for the efficacy of the proposed organizational model.

**63. Egnew, T.R. The Meaning Of Healing: Transcending Suffering. The Annals of Family Medicine 2005, 3, 255–262. https://doi.org/10.1370/afm.313**	Qualitative inquiry into the meaning of healing. Explores healing as a response to suffering. Supports the possibility of meaningful recovery beyond cure.	Interpretive work in a particular professional context. Cannot be generalized as a universal definition or effect estimate.

Table S3 From Published Findings to Candidate Mechanisms

The categories and loop were provisionally formulated from professional experience and preliminary reading. This table explains how published findings support or constrain the present formulation. It does not reconstruct a dated development log or imply independent inductive coding. The cited theories provide interpretive lenses, while the three-part classification is the authors' proposal.

Candidate Mechanism and Conceptual lens	Supporting Findings and Counterarguments	Qualification Adopted in the Framework
**Representation and design. Organizational decoupling [8,9].**	Collective participation studies identify barriers to influence [11,52]. Successful implementation accounts describe organizational change [30–32]. Existing person-centred frameworks already connect structures, processes, and outcomes [3,4].	The mechanism concerns substantive consideration of contributions, not whether every proposal is accepted. Collective design and individual use need separate evidence. Neither standardization nor plan retention proves a loss.
**Voice and agency. Relational autonomy and epistemic injustice [54,56].**	Studies identify fear, deference, and other constraints [14–16,33]. Patients may also prefer supported delegation. A vignette trial measures willingness to speak, not actual influence [55].	A changed account is a signal, not proof of earlier suppression. Establish timing, initial safety, and new information. Respect chosen delegation and retain uncertainty.
**Measurement and response. Means–ends decoupling applied to feedback [9].**	PREM reviews identify incomplete evaluation of organizational responses [12,13]. PROM studies show mixed and sometimes positive effects, including quality of life benefit [18–22].	The claim concerns information left unconsidered when a response is indicated. Justified retention counts as a response. Positive findings rule out general claims that measurement fails. No mandatory architecture or review interval has been established.

Table S4 Proposed Operational Coding Rules

These rules are a starting point for prospective development. They have not been validated. The unit is an individual priority within a defined care episode and observation period. More than one mechanism can be assigned to an episode, but each requires its own evidence. An unclassified category is retained for material that does not fit the framework.

Property or Decision	Evidence and Coding Rule	Alternative Explanation or Safeguard
**Patient priority and its timing**	Retain the person's wording where feasible, jointly clarify meaning and record when the priority formed or changed. Record desired participation and any proxy contribution separately.	Do not treat a group average, a physiological sign, or family preference as the patient's unspoken wish. New information can change priorities.

**Candidate design mechanism**	Look for evidence that a relevant patient contribution was not substantively considered in options, rules, or resources. Trace any collective proposal to implementation and individual availability.	Declining a proposal with a considered rationale is not equivalent to exclusion from consideration. An option may already address the concern.
**Candidate voice mechanism**	Seek corroborated evidence that a pre-existing priority was withheld or disregarded because expression felt unsafe or ineffective. Ask why the account changed.	Protected interviews are not a gold standard. Use timing, observation, and appropriate repeat-measurement comparisons. Stable answers may reflect initial safety.
**Candidate response mechanism**	Establish that relevant information reached the responsible team and whether consideration, indicated action, and review followed within a suitable period.	A justified decision to retain treatment is a response. Delay must be interpreted against urgency and a prespecified observation period.
**Justified retention or modification**	Code the rationale, the patient position considered, and the care delivered. Keep clinical limitations distinguishable from a plan that already fits the person's goals.	Concordance does not require every request to be met. A patient may understand and still disagree with a clinical limitation.
**Insufficient evidence**	Use when available records, observation, and accounts do not establish what was considered or done. Preserve conflicting accounts for analysis.	Do not recode missing documentation as failure or success. Missingness may differ across settings or patient groups.
**Reliability and overlapping categories**	Use independent assessors and a dated manual. Report mechanism-specific agreement before adjudication, uncertainty intervals, co-occurrence, and unclassified cases. Examine equivalence across languages and settings.	Do not combine overlapping labels into an unvalidated total score. Keep training examples separate from the reliability sample. Each label needs its own evidence.
**Intervention effect and fidelity**	Verify actual delivery and control team responses, timing, clustering, and baseline differences. Address observation-related attention and missing data. Define a meaningful effect before comparison.	A non-significant result or overlapping group confidence intervals alone does not establish equivalence. Analyze uncertainty in the between-group contrast. Do not reduce fidelity to completed fields.
**Actual care and burden**	Assess the delivered response and the patient account separately from completeness of recording. Measure documentation time, care time, access, safety, and family work.	Avoid rewarding more records without more informative care. Reuse existing information and targeted samples before adding routine forms.

An optional cumulative question bank would need patient involvement, cognitive testing, and content validity assessment. Test any clustering on new material and retain rare or unclassified concerns. Clusters may inform service planning, but cannot determine an individual's priorities. The bank would support elicitation, not establish that a priority influenced decisions and delivered care.
